# Thanks to Our Reviewers in 2022

**DOI:** 10.1093/abt/tbad005

**Published:** 2023-03-24

We would like to take this opportunity to express our sincere appreciation for those who reviewed manuscripts for our journal *Antibody Therapeutics*. The valuable insights and feedback from our reviewers are essential in maintaining the high standards of our journal for serving the scientific community.

We recognize that reviewing manuscripts is a demanding task that requires a significant amount of time and effort, and we are truly grateful to the reviewers for their dedication and commitment to this important role. Their generosity in sharing expert knowledge and expertise has been invaluable. We look forward to continued support and collaboration as we work together to advance the field of antibody engineering and therapeutics.

The experts listed below, as well as a number of other reviewers who would like to remain anonymous, reviewed manuscripts for *Antibody Therapeutics* in 2022.

Ab, Olga

Akiba, Hiroki

An, Zhiqiang

Angkasekwinai, Pornpimon

Antignani, Antonella

Bhatia, Hema

Booth, Brian

Borth, Nicole

Carter, Paul

Chen, Chun

Chen, Lei

Chen, Xiaocheng

Chen, Xin

Chen, Yuanzhi

Chen, Yuxin

Chen, Zhoujiang

Chen, Zirong

Cho, Yong Ku

Chow, Sih-Yao

Chuang, Ray

Chung, Junho

Feng, Mingqian

Feng, Zijie

FitzGerald, David

Fort, Madeline

Fu, Ying

Gao, Wenda

Gavin, Marc

Ge, Xin

Goswami, Arvind

Gui, Xun

Gurusamy, Balakrishnan

He, Xin

Hirasawa, Noriyasu

Huang, He

Huang, Jian

Huang, Lei

Jiang, Weidong

Jung, Sang Taek

Karunasagar, Iddya

Kathuria, Sagar

Klein, Christian

Ku, Sherman

Ku, Zhiqiang

Laber, Joshua

Li, Nan

Li, Shichang

Li, Shuqi

Li, Wei

Li, Xinxin

Liu, Dayou

Liu, Xiaoguang

Lou, Yang

Ma, Ningning

Malm, Magdalena

Meng, Feilong

Morris, Christopher

Narhi, Linda

O’Mara, Brian

Ou, Jianfa

Pelegri-O’Day, Emma

Pirhanov, Azady

Polson, Andrew G.

Prior, Sandra

Qiu, Yu

Rader, Christoph

Rai, Praveen

Raina, Deepak

Ratnam, Manohar

Rosenthal, Dean

Sarangapani, Prasad

Shen, Guodong

Sun, Yaping

Tustian, Andrew

Vasquez, Maximiliano

Wang, Ziming

Williams, John

Xia, Ningshao

Xu, Jianqing

Yu, Xin

Zhan, Jinbiao

Zhang, Sheng

Zhang, Wei

Zhang, Xiao

Zhang, Xuyao

Zhang, Yang

Zhao, Zhihui

Zong, Shuai

